# 

**Published:** 1897-11-06

**Authors:** 


### EARLY EDITION.

**Notices of Births, Deaths, and Marriages** are inserted in this column at a charge of 2s. 6d. for an announcement not exceeding four lines.

DEATH.

On October 28th, at the Infirmary, St. George-in-the-East, of enteric fever, Mary Dawson (nurse), aged 34 years. "Until the Day dawn and the Shadows flee away." (1,103)

### NOTICE TO CORRESPONDENTS.

All MS., Letters, Books for Review, and other matters intended for the Editor should be addressed The Editor, "The Hospital," The Hospital Building, 28 & 29, Southampton Street, Strand, W.C.

The Editor cannot undertake to return rejected MS., even when accompanied by stamped directed envelope.

**Notice to Advertisers and Subscribers.**

All Advertisements, Orders for Copies of The Hospital, and Business Communications should be addressed to **THE MANAGER, (Not to The Editor),** The Hospital, The Hospital Building, 28 & 29, Southampton Street, Strand, London, W.C.

The Cost of Subscription, post free, is as follows:—Per week, 2½d.; per quarter, 2s. 8d.; per half-year, 5s. 3d.; per annum, 10s. 6d. Foreign:—Per annum, 15s. 2d.; payable, in all cases, in advance.

**NOTICE.—Vol. XXII. of "The Hospital" (April, 1897, to September, 1897), in handsome cloth binding, is now on sale at the Office of this Journal, price 7s. 6d. Cases for binding the Numbers contained in this Volume, 1s. 6d. Index to Vol. XXII., 2½d., post free.**

**The Monthly Part for November is now ready, Price 6d., by post 8d.**

### SCRAPS AND GLEANINGS.

The Portsmouth Jubilee Fund for the rebuilding of the hospital amounts to £15,500.

The new women's ward at the Chester Infirmary has been formally opened.

An inquiry into the present state of the finances of Addenbrooke's Hospital, Cambridge, is to be held.

### The Student of Medicine.

### APPOINTMENTS.

P. Coleman, M.B., B.S.Durh., M.R.C.S.Eng., L.R.C.P.Lond., appointed Honorary Medical Officer to the Middlesex Hospital Convalescent Home, Clacton on Sea. T. Coleman, M D., C.M.-Toronto, appointed a Clinical Assistant to Out-patients at the Chelsea Hospital for Women. E. Cottew-Moore, M.B & M.S.-Edin., appointed Senior House Surgeon to the Birmingham and Midland Eye Hospital. M. G. Dyson, appointed Resident Second Assistant Medical Officer to the Workhouse and Infirmary of the Islington Union. Dr. N. Ebrington, appointed Medical Officer for the Wetherfield District of the Braintree Union. J. J. Evans, M B. & M.S.Edin., M.R.C.S.Eng., appointed Resident Surgical Officer to the Birmingham and Midland Eye Hospital. H. B. Gardner, M.R.C.S.Eng., L R.C.P.Lond., appointed Anæsthetist to the National Orthopædic Hospital. J. E. Gemmell, M.B., C.M.Edin., appointed Honorary Assistant Medical Officer to the Hospital for Women, Shaw Street, Liverpool. T. Gregory, M R.C.S., L.R.C.P., appointed Second Assistant Medical Officer of the Crumpsall Workhouse of the Township of Manchester. F. Hawthorn, M.D.Durh., B.S., appointed Medical Officer for the No. 4 District of the Newcastle-upon-Tyne Union. J. L. Johnstone, M.R.C.S., L.R.C P., appointed Medical Officer of Health to the Upholland District Council. W. Macdonald, L R C.S., L.R.C.P.Edin., appointed Senior House Surgeon to the Royal Albert Edward Infirmary and Dispensary, Wigan. S. C. H. Moberley, M.R.C.S., L.R.C.P.Lond., appointed Medical Officer for the Second District of the Winslow Union. J. E. Moorhouse, M A., B.Sc., M.D., appointed Medical Officer to the Stirling Combination Poorhouse. G. H. Parry, M.B., C.M.Edin., appointed Resident Physician to the Victoria Hospital for Consumption and Diseases of the Chest, Craigleith, Edinburgh, and also Non-Resident Clinical Clerk to the Observation Ward, Royal Infirmary, Edinburgh. G. H. Philipson, M.D., D.C.L., F.R.C.P., appointed Medical Visitor to the Dunstan Lunatic Asylum. C. M. Phillips, M.D.Brux., M R.C.S.Eng., L.R.C.P Lond., appointed Surgeon to the Bristol Dispensary. J. J. Redfern, M.A., M.D., M.Ch., M.A.O I., appointed Honorary Medical Officer to the Croydon General Hospital. J. C. Renton, M.D.-Edin., L.R.C.S., appointed Visiting Surgeon to the Western Infirmary, Glasgow. R. Stockman, M.D., F.R.C.P.Edin., appointed Visiting Physician to the Western Infirmary, Glasgow. H. E. Thompson, M.B.Lond., M.R.C.S.Eng., appointed Junior House Surgeon to the Birmingham and Midland Eye Hospital. H R. Todd, L.R.C.P., L.R.C.S I., appointed Medical Officer for the No. 10 District of the Croydon Union. F. H. Waddy, M.D.Glasg., M.B., C M., appointed Assistant Medical Officer to the Sheffield Union Workhouse. J. J. Wadelow, F.R.C.S.Eng., L.R C P.Lond., appointed Medical Officer for the Workhouse and the Southern District of the Whittlesey Union. R. S. Wells, L R.C.P. & S.Edin., L.F.P.S.Glasg., appointed Medical Officer for the Parish of Kirkcowan, Wigtownshire. A. Wilsden, M B., C.M., appointed Clinical Assistant to Out-Patients at the Chelsea Hospital for Women. C H. Moorhouse, M.B., Ch.B., appointed Resident Obstetric Officer to the General Infirmary, Leeds. N. G. Meade, M.R.C.S., L.R.C.P., appointed House Physician to the General Infirmary, Leeds. R. S. Collier, M.R.C.S., L.R.C.P., appointed House Physician to the General Infirmary, Leeds. J. F. Dobson, M.R.C.S., L.R.C.P., appointed House Surgeon to the General Infirmary, Leeds. J. V. Hartley, M R.C S., L.R.C.P., appointed House Surgeon to the General Infirmary, Leeds. H. Veale, L.S.A., appointed Resident Medical Officer to the Ida Hospital, Leeds.

